# Tree Cover Bimodality in Savannas and Forests Emerging from the Switching between Two Fire Dynamics

**DOI:** 10.1371/journal.pone.0091195

**Published:** 2014-03-24

**Authors:** Carlo De Michele, Francesco Accatino

**Affiliations:** Department of Civil and Environmental Engineering, Politecnico di Milano, Milano, Italy; Georgia State University, United States of America

## Abstract

Moist savannas and tropical forests share the same climatic conditions and occur side by side. Experimental evidences show that the tree cover of these ecosystems exhibits a bimodal frequency distribution. This is considered as a proof of savanna–forest bistability, predicted by dynamic vegetation models based on non-linear differential equations. Here, we propose a change of perspective about the bimodality of tree cover distribution. We show, using a simple matrix model of tree dynamics, how the bimodality of tree cover can emerge from the switching between two linear dynamics of trees, one in presence and one in absence of fire, with a feedback between fire and trees. As consequence, we find that the transitions between moist savannas and tropical forests, if sharp, are not necessarily catastrophic.

## Introduction

The tree cover distribution of moist savannas and tropical forests has been recently investigated extensively over Africa, Australia and South America [1], [2]. [1] have shown that these biomes co-exist over a very large range of annual rainfall (650–2500 mm/yr) and that at intermediate rainfall (1000–2500 mm/yr) tree cover is bimodal, exhibiting one peak for low woody cover (savanna - characterized by high fire frequency) and one peak for high woody cover (forest - characterized by absence of fire).

The bimodality of tree cover distribution has been used as a proof that savanna and forest are alternative stable states [1]–[3]. In literature, many simple models (also denominated minimal) exhibit savanna–forest bistability [4]–[6]. The main advantage of minimal models consists in the possibility of identifying interactions and causal *nexi* between drivers and state variables [7], [8]. These models can be treated analytically and investigated through the bifurcation analysis. The savanna–forest models of [4]–[6] are systems of non-linear differential equations, forced by fire and other environmental factors such as rainfall (without exploring its variability though) and herbivores. What is widely accepted is that fire is responsible for bistability: in savanna and forest the tree cover cannot be adequately explained without explicitly considering the dynamics of fire [9]–[12], and feedbacks fire–trees have been used in literature to argue for bistability (see [13] and references therein).

The literature contains many studies about the existence of alternative stable states (see the reviews by [13], [14]) and models exhibiting bistability [15]–[17]. However, alternative stable states have been more frequently found in laboratory experiments than in field studies, even after correcting for different number of studies [13]. Bistability has deep ecological consequences on the ecosystem behavior: the existence of critical transitions, which makes the ecosystem fragile around certain thresholds, subject to catastrophic shifts, and thus difficult to manage.

We believe that the bistability of savanna and forest is not the only possible explanation for the bimodality of tree cover distribution. Here we use a minimal model (the same model typology used in literature to prove savanna–forest bistability [4]–[6]) to provide an alternative explanation to tree cover bimodality. We focus on the representation of fire, which is the key element explaining the bimodality of frequency distribution. In the above mentioned non-linear differential equation models, fire is represented as an a-priori determined parameter. Here we consider the vegetation subject to two possible dynamics: one in presence and the other in absence of fire. The switching between these two dynamics is stochastic, and dependent on the quantity of trees in the ecosystem.

Section *Methods* gives the methodology. In particular, firstly it illustrates the matrix model of tree dynamics in moist savannas and tropical forests, then it shows how the model can be reduced to a Markov chain, and reports the calculation of the stationary probability distribution of the tree cover. Section *Results* provides three examples of how tree cover bimodality can be found, including a sensitivity analysis of parameters. Section *Discussion* comments the results and provides some considerations to support the alternative explanation of tree cover bimodality in savanna–forest dynamics.

## Methods

Let us assume the yearly dynamics of trees in moist savannas and tropical forests described by a time-discrete matrix model. This choice is motived by two facts: 1) matrix models are dominant in plant demography description, have an important role in studies of broad ecological and evolutionary questions [18], and the parameters determination is relatively easy [19], [20], as well as the comparison of results obtained using different size or age classes discretization [18]. 2) Matrix models have already been considered to describe the tree dynamics in savannas by [21]–[23]. In these studies, fire occurrence is assumed independent of the state of the system. In particular [22], use a deterministic fire return period [21], assume a constant probability of fire occurrence [23], considers both these approaches. However, feedbacks between fire and vegetation have been observed [9], [10]. In order to account this element within the vegetation dynamics, we consider fire as a stochastic process with a probability of occurrence dependent on the state of the system.

Let 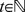 be the temporal coordinate, 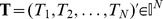 the 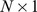 vector representing the fraction of tree cover divided in size classes: 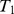 represents the seedlings, and 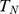 represents adult trees. The superscript 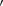 stands for transposition, and 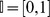. Each component of 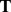 is dimensionless, with 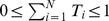.

The yearly dynamics of 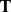 are described by the following matrix equation:
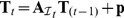
(1)where 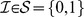 is a *Bernoulli* variable describing fire occurrence. 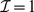 is associated to the tree dynamics in presence of fire, and 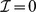 to the tree dynamics without fire. For each year 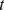, 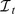 takes value 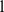 with probability of fire 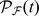, and 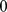 with probability 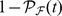. 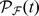 depends on the status of the ecosystem, *i.e.*, by the amount of grass fuel load, which is negatively correlated to the density of trees [24]–[26]. In particular, if there are fewer trees, there is more grass in moist savannas, and thus a greater quantity of fuel load enhancing fire probability [27], [28]. Thus we assume a negative relation between tree density and fire probability, considering only implicitly the negative correlation between grass density and tree density. This implies a *positive feedback*, which means that the more the ecosystem burns, the more it is likely to burn in future [9], [10]. The temporal sequence 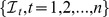 is a two-state *Markov chain* with a 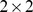 transition matrix 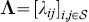, where 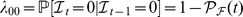, 
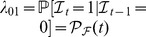
, and similarly 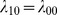 and 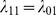. The 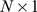 vector 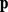 represents the recruitment. Assuming that new seedlings are recruited in the smallest size class, 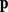 has the first element 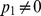 (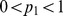), and 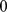 elsewhere. 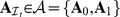 is a 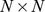 matrix. In particular 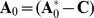, 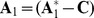, and 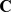 is a 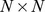 matrix containing in the first row all elements equal to 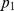, and 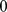 elsewhere. 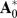 and 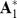 are Lefkovitch matrices. According to Eq.(1), in each temporal step, the trees are firstly updated according to absence/presence of fire, and then new tree seedling are recruited in the population. Thus the fire can affect the new recruits the following years.

The generic element 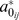 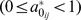 of 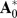 represents the proportion of trees of class 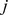 moving into class 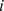 in the year 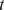 if the fire does not occur, while the generic element 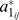 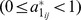 of 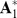 represents the proportion of trees of class 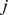 moving into class 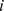 in the year 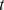 if the fire occurs.

According to Eq.(1), the annual variability of trees comes out by the composition of two linear dynamics, one in presence, and the other in absence of fire. Eq.(1) is also known in literature as *Hybrid Linear System*, or *Markov Jump Linear System*, see [29]. The switching between the two dynamics is stochastic, and ruled by the fire probability 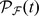, which is dependent on the amount of trees in the ecosystem, 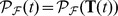. Let 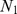 indicate the number of visits the system makes in the 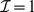 dynamics, *i.e.*, the number of years when the fire occurred, while 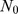 the number of visits in the 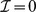 dynamics, *i.e.*, the number of years without fire. The *average* 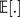 number of visits in the 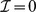 dynamics over a period of 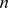 years is calculated as 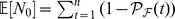, and similarly for the 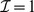 dynamics 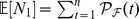. Thus the permanence ratios 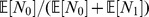 and 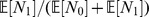 indicate respectively the percentage of time the system spends in no-fire and fire dynamics.

For Eq.(1), two possible steady states 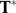 can be found. They are summarized in the following equation:
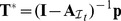
(2)where 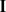 is the identity matrix. By putting 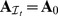, the steady state 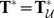 is found. This corresponds to the *undisturbed* steady state, and it is obtained if the matrix 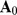 is always applied, *i.e.*, the system is always in no-fire dynamics. Analogously, putting 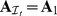, the steady state 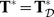 is found, and corresponds to the *disturbed* steady state, which is obtained if the matrix 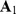 is always applied, *i.e.*, the system is always in the fire dynamics. Note that each of two steady states is reached if the system stays in one dynamic only (or, let's say, for a certain amount of time). However, the global dynamic of 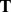 generally oscillates between these two steady states, resulting in non-equilibrium dynamics.

The system of Eq.(1) is *asymptotic stable*, *i.e.*, it does not go to infinity, if the product of all possible sequences of matrices 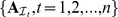 leads to an asymptotically stable solution, *i.e.*, tends to zero, see [30], [31]. A sufficient stability condition for this is that 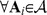, 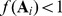, where 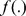 is any *matrix norm* function [30]. As matrix norm it is possible to consider the *maximum element norm* [32]. Here, the conditions 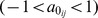 and 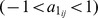 ensure the asymptotic stability of Eq.(1).

Eq.(1) is a time-discrete *Markovian* process, which for simplicity of analysis, we represent through a finite-state *Markov chain*, *i.e.*, with discrete and finite states, discretizing the domain of the variable. Let the domain of each component of 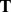 be divided into 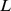 intervals of width 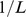. Thus the domain of 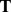 (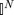) is discretized into 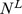 hypercubes, where the 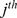 element is identified through its center of coordinates 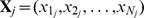. Of the 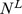 hypercubes, some of these have no points satisfying the condition 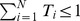, thus these elements are not physically acceptable. Let be 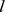 the number of physically acceptable hypercubes. Let 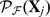 be the fire probability of the hypercube 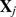, evaluated in its center. Each hypercube can be considered as the state of a Markov chain. Let 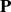 be the 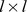 transition matrix associated to the Markov chain, where the generic element 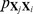 contains the probability that the system passes from the state 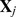 to the state 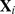. Let 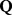 be the 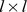 transition matrix containing in each element 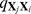 the proportion of points of the hypercube 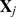 mapping into the hypercube 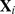 when the dynamics of 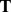 are ruled by the no-fire dynamics, and 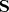 be the 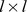 transition matrix containing in each element 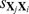 the proportion of points of the hypercube 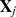 mapping into the hypercube 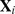 when the dynamics of 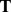 are ruled by the fire dynamics. For the theorem of total probability, 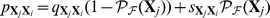. Let 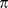 be the 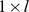 vector representing the stationary distribution of the states of the chain, whose elements are non-negative and sum to 1. The stationary distribution satisfies the equation 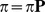, or equivalently 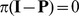. If the Markov chain is irreducible and aperiodic, then the stationary distribution is unique. In this case the 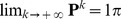 converges to a rank-one matrix in which each row is the stationary distribution. The distribution 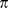 will have a *bimodal* or *unimodal* shape depending by the mixing of the two dynamics, fire and no-fire. In particular, if the system persists in a single dynamic 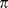 will be unimodal, otherwise it will have a bimodal, or unimodal shape, depending on the value of the permanence ratios.

## Results

Here we give three examples of tree cover bimodality emerging from the switching between fire and no-fire dynamics.

As first example, we consider the simplest case 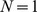, *i.e.*, 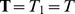, the dynamics of tree cover is described only by one size class. The matrices 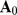 and 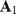 and the vectors 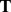 and 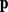 are scalar and therefore for this case are not shown in bold. We consider this case to limit the number of parameters to five, nevertheless the conclusions we draw apply also to 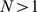, reasoning in terms of total tree cover.

**Figure 1:**
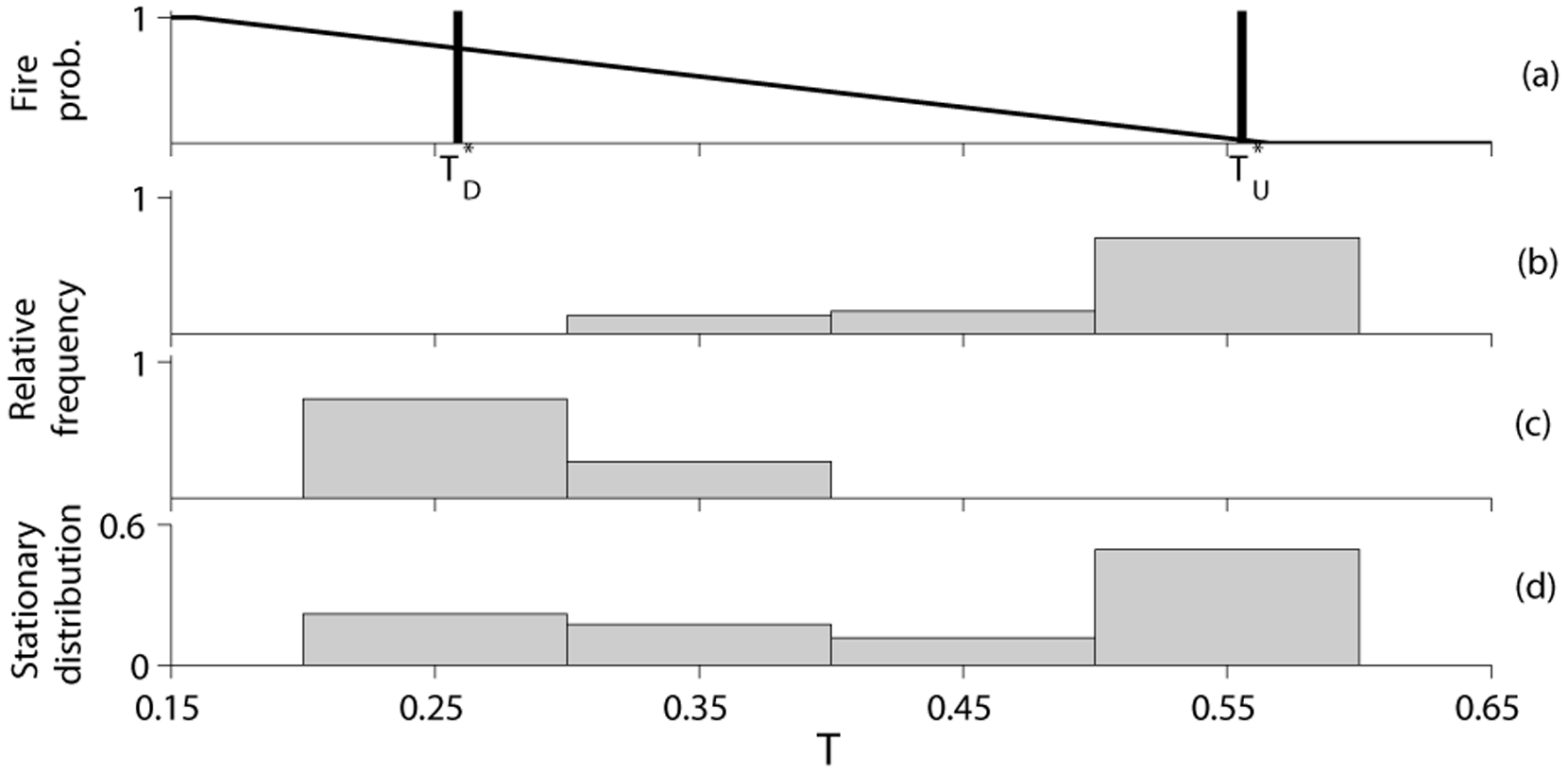
Example of tree cover distribution. Panel a) reports the fire probability, 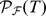, with the indication of the steady states of tree cover: 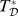 and 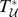. Panel b) shows the histogram of tree cover when fire is not present. Panel c) shows the histogram of tree cover when fire is present. Panel d) gives the stationary state distribution 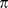 of tree cover. In panels b–d, bins of 0.1 width are used.


The fire probability, 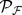, is assumed a decreasing linear function of the tree cover 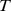. In particular 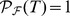 for 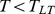, 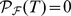 for 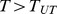, and 
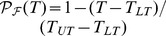
 for 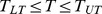, where 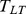 and 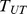 are two thresholds (Fig. 1a). The choice of the shape of 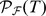 results from two arguments: 1) the fire probability may be assumed to be a linear increasing function of dead grass biomass as in [11], [12], [33] and according to field data showed in [34]. 2) Grass production increases as the tree cover decreases [24]–[26].

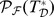
 is the probability the ecosystem stays in the state 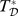, whereas 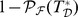 represents the risk of the ecosystem to leave 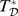. Similarly, 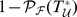 represents the probability of staying in 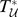 and 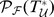 is the risk of leaving 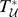. If, for instance, 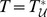 and 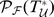 is very close, but different from zero, the ecosystem tends to stay in the undisturbed state. However, because the probability of fire is not zero, a fire event may happen. If this occurs, the ecosystem reduces its tree cover, and at same time it increases the probability of fire. In other terms, the more the ecosystem burns and more likely it is likely to burn in the future [10]. Analogously, if 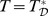 and 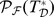 is very close, but different from unit, the ecosystem tends to stay in the disturbed state, because fire is likely to occur every year. However, because the fire probability is not one, in a certain year, the fire event could not occur. In this case, in the following year the ecosystem will increase its tree cover, and at same time reduce the probability of fire. Thus, if the ecosystem does not burn in a year, it is unlikely to burn the following year [9], [10]. The above reported function 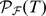 includes the feedback between fire and tree cover.

Let us assume for the five parameters the following values: 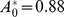, 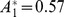, 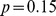, 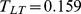, and 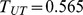. These values are calculated with transition matrices of *Acacia nilotica* [21] using the collapsing algorithm proposed by [19]. 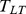 corresponds to a grass fuel load of 1000 kg/ha, value under which 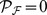; 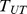 to a grass fuel load of ∼11000 kg/ha, values above which have 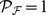, see [33], [35]. The stable states of the two dynamics are 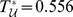, and 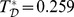 with fire probabilities respectively 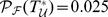 and 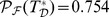. We have made a 5000 yr simulation of the model starting from the initial condition 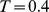. It is important to make simulations on long time horizons (millennia) in order to avoid partial representations of the ecosystem behaviour [36], [37]. The permanence ratios in the two dynamics are respectively 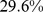 in the no-fire dynamic, and 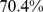 in the fire dynamic. The histograms of tree cover evaluated with bins of 0.1 width are reported in Fig. 1: the histogram of the no-fire dynamics has a mode around the state 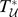 (Fig. 1b) and the histogram of the fire dynamics has a mode around the state 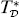 (Fig. 1c). Then, we have discretized the variable 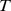 using bins of 0.01 width, calculated the Markov transition matrix 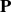, and consequently the stationary probability distribution 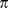, given in panel d) of Fig. 1. From this, it is evident that the stationary distribution of tree cover is bimodal.

**Figure 2:**
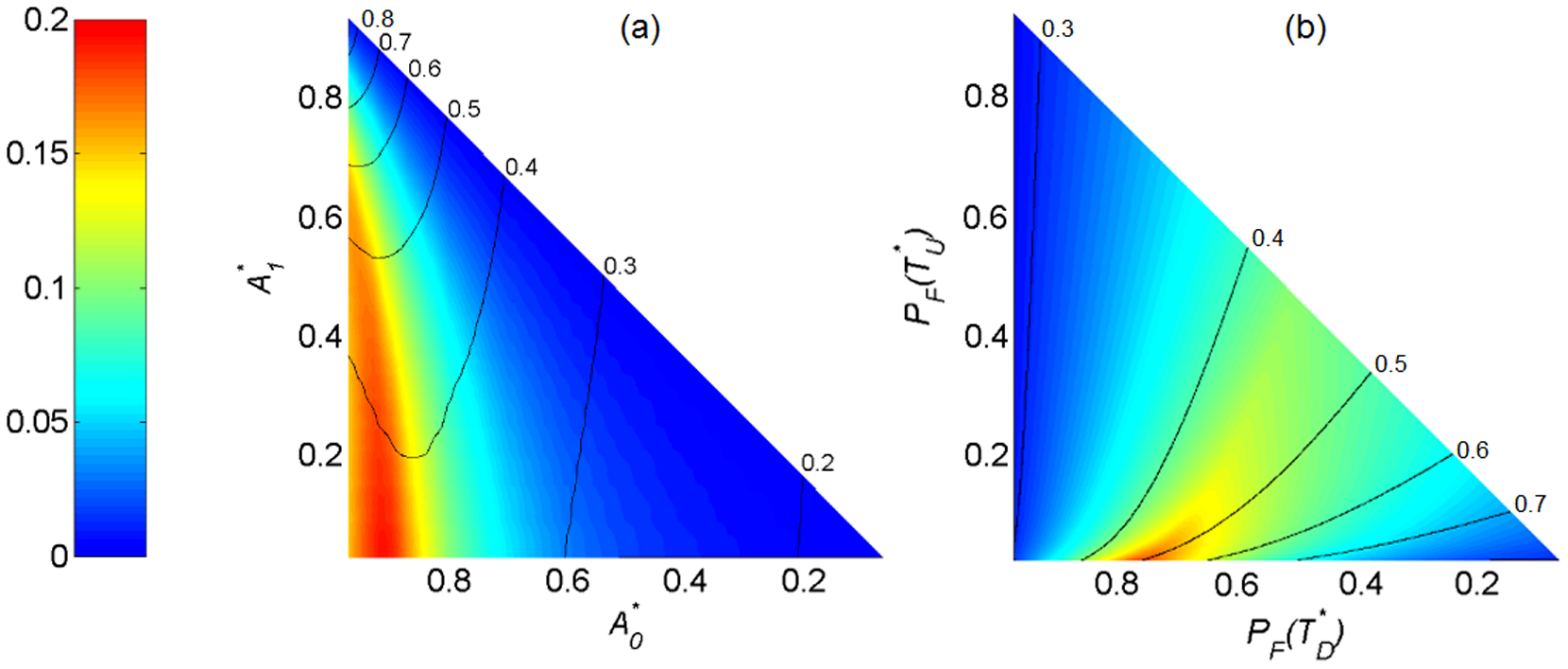
Sensitivity analysis of the shape of the probability distribution 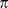 of tree cover using the bimodality index 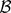. In panel a) the demographic parameters 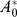 and 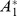 are varied. In panel b) the parameters of fire probability 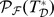 and 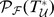 are varied. The black lines represent 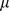-isolines.

Clearly, this is only an example, and the results are dependent on the specific parametric configuration. A sensitivity analysis of parameters can clarify the circumstances under which a bimodal distribution of tree cover can emerge. In order to quantify the bimodality of the tree cover distribution, we introduce here a *bimodality index* 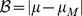, where 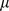 is the mean of the distribution 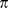, while 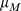 is the mean of 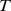 associated to the more frequent dynamic. To estimate 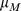, we built a histogram of the values associated to the more frequent dynamic with the same bins of the 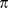 distribution. Then 
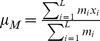
, where 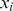 is the center of the 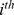 bin, 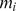 the absolute frequency of the 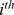 bin. If 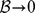 then the distribution is *unimodal*. If 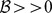 then the distribution is *bimodal*. We found that 0.1 can be used as threshold to discriminate between unimodal and bimodal shape. We have then considered the index 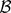 to analyze the shape of 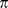 firstly varying the demographic parameters, 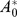, and 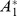, and keeping the other parameters constant as in the example previously reported, and secondly varying the parameters of the fire dynamics 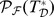 and 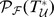, *i.e.*, adjusting the thresholds 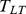, and 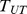, and keeping the other parameters constant as in the example. In panels a) and b) of Fig. 2 we report the results of the sensitivity analysis. Because the demographic parameters must satisfy the constraint 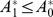, and similarly for the parameters of the fire dynamics 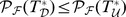, then the graphs in Fig. 2 are triangular. In the panels of Fig. 2 we also report the isolines of 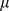, so that in the case of unimodal distribution we have information about the location of the histogram peak.

If 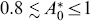 and 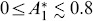, then the distribution is bimodal, otherwise it is unimodal (Fig. 2a). If 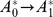, *i.e.*, at the top and right vertices of the triangle, then the two dynamics tend to collapse into one, and the distribution 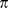 is unimodal. In particular, if 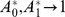, 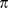 is located in the right part of its variability range (*i.e.*, high values of the tree cover), while if 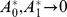, 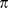 is located in the left part of its variability range (*i.e.*, small values of the tree cover).

For 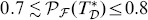 and 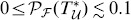, the distribution is bimodal, otherwise it is unimodal (Fig. 2b). For small values of 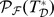, and consequently 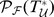, the ecosystem is seldom disturbed by fire, and is characterized by high values of the tree cover. The distribution 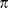 is unimodal and located in the right part of its variability range. Conversely, for high values of 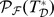, and 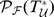, the ecosystem is frequently disturbed by fire and is characterized by low values of tree cover. The distribution 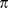 is unimodal, and located in the left part of its variability range.

**Figure 3:**
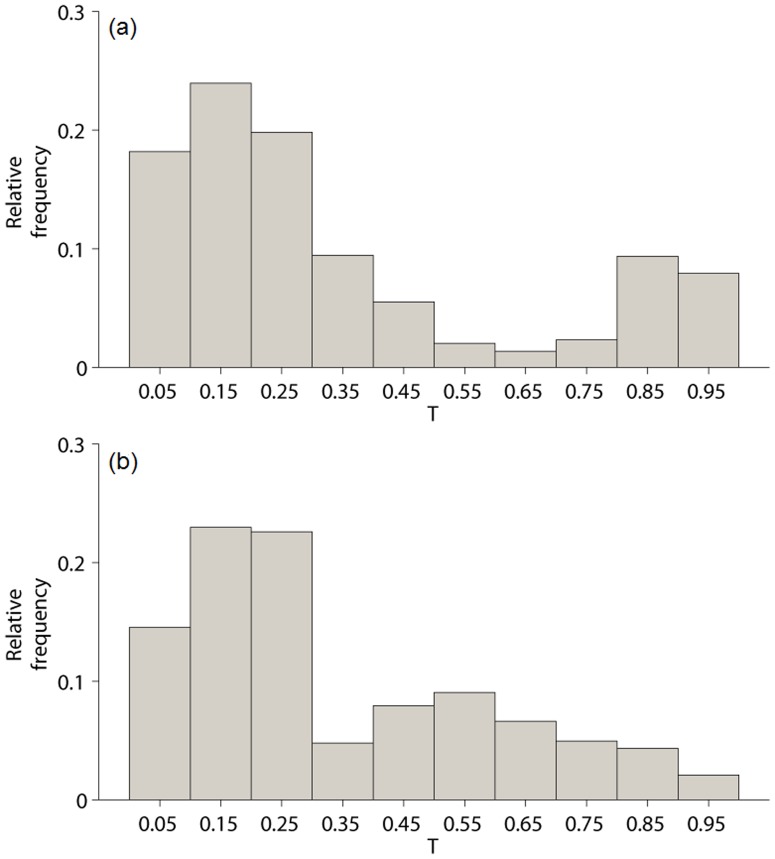
Frequency distribution of tree cover obtained sampling from 1000 simulations, each one 5000 yr long, at the time t = 300 yrs. In panel a) the 1000 simulations are obtained uniformly sampling the parameters from the following intervals: 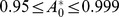, 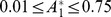, 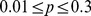, 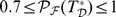, and 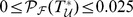. In panel b) from the following intervals: 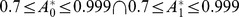, or 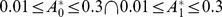, 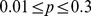, 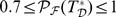, and 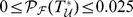. Bins of 0.1 width are used.

As second example, we mimic a spatial sampling of tree cover. In literature [1], [2] the tree cover distribution is empirically obtained collecting data at different points in space, and at a certain time instant, rather than sampling the data at a given location over time, *i.e.*, using the spatial information in place of the temporal one. In order to mimic the spatial sampling of tree cover from distinct and independent sites at a given instant time, we have made 1000 simulations, each 5000 yr long with 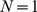 using for each simulation parameter values extracted uniformly from the following intervals: 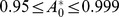, 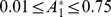, 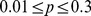, 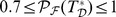, and 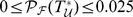. We have adopted narrow ranges for the parameters associated to the no-fire dynamic, *i.e.*, 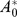 and 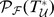, and wider variability ranges for the parameters associated to the fire dynamic, *i.e.*, 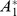, and 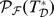, as well as the recruitment parameter 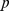, in this way, we include different fire vulnerability of different tree species [23]. Depending on the evolutive strategies of trees, there are trees very resistant to fire with a thick bark or resprouting mechanisms (savanna trees), and there are trees very vulnerable to fire (forest trees). Conversely, the mortality of trees without fire is far less variable. Note that the intervals chosen for 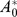 and 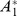 correspond to bimodal distributions of tree cover (Fig. 2a). Sampling the tree cover from the 1000 simulations, in any time instant (except for the first 100 yrs possibly influenced by the initial state), we have found a bimodal distribution, with values of the bimodality index 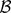 in the range 0.1–0.23. Panel a) of Fig. 3 reports the empirical distribution of tree cover sampled at 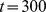 yrs.

We have also sampled tree cover using parameters extracted uniformly from the following intervals: 
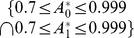
, or 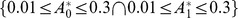, 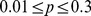, 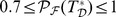, and 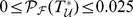. Note that the intervals chosen for 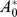 and 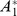 correspond to unimodal distributions of tree cover (Fig. 2a). In particular, the configurations belonging to 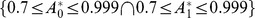 are characterized by high values of tree cover, while configurations belonging to the range 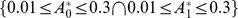 by small values of tree cover. The frequency distribution of tree cover obtained from the spatial sampling is however bimodal in any time instant (except for the first 100 yrs possibly influenced by the initial state), with values of the bimodality index 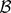 in the range 0.18–0.2. Panel b) of Fig. 3 gives the frequency distribution of tree cover sampled at 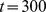 yrs.

**Figure 4:**
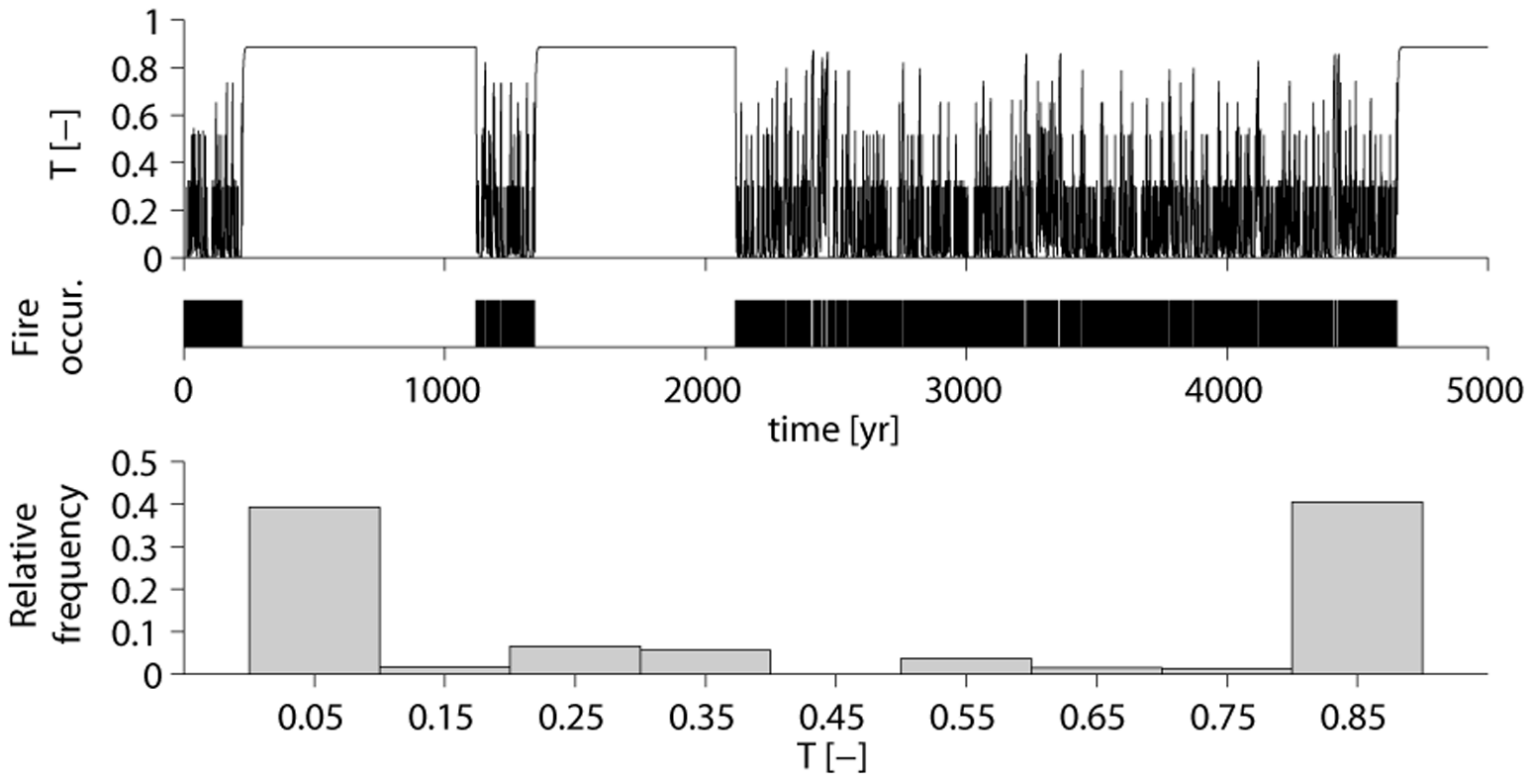
Tree cover dynamics (panel a), fire occurrence (panel b), and probability distribution of tree cover (panel c) of a tree population classified in three height classes. In panel c), bins of 0.1 width are used.

As third example, we consider 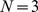, *i.e.*, a tree population classified in three size classes. In particular, class 1 includes small trees (*i.e.*, height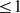 m), class 2 medium trees (*i.e.*, 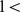 height 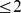 m), and class 3 large trees (*i.e.*, height 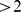 m). The matrices 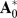, 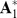 and the recruitment vector 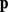 are respectively equal to 
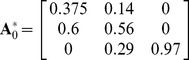
, 
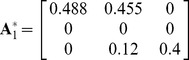
, 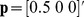. These values are derived from [33] passing from the seasonal matrices to the annual ones. The parameters 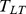 and 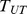 of the fire probability refer to the tree cover calculated over the last two classes, *i.e.*, 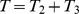, assuming that the first height class does not contribute significantly to the vegetation cover. As in [33], we use 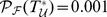 and 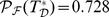, and as initial condition 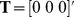. Figure 4 shows in the top panel a 5000 yr simulation of the tree cover, in the intermediate panel the occurrences of fire, and in the bottom panel the stationary probability distribution of the tree cover.

## Discussion

The three examples show how the model in Eq.(1) can exhibit the tree cover bimodality under different ways. For certain parameter combinations, a double peak could be shown in the frequency histogram of the states visited along a trajectory over time (first and third example), or equivalently, in the stationary state probability distribution of the Markov chain associated to the system (first example). Bimodality of tree cover has also been obtained in the frequency histogram of the states visited by many simulations (each one representing a different site) at a certain time instant (second example). In all these cases, bimodality simulated through the model is not in contradiction with bimodality observed in data [1], [2] and it does not involve the concept of bistability.

The first example, showing tree cover bimodality in a single non-equilibrium trajectory along time, is in agreement with some statements of [13], [38] according to which data may not necessarily represent steady states, but may be snapshots of the system which is not at the steady state.

The sensitivity analysis explains in which parametric configurations bimodality can be obtained in the trajectory. In the first two examples, the demographic parameter 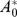 is representative of how fast the woody cover grows in absence of fire. The higher this parameter is, the more the tree dynamics without fire tend towards the undisturbed state 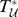. The parameter 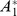 is representative of the resistance of the tree vegetation to fire disturbance. The lower this parameter is, the more attractive are the tree dynamics with fire towards the state 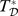. If trees are fast growing and highly resistant to fire (i.e, the top corner of Fig. 2a), the woody cover tends to be unimodal with a high average value (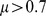). If trees are slow growing with a low fire resistance (*i.e.*, the bottom right corner of Fig. 2a), the woody cover tends to be unimodal with a low average value (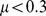). Bimodality occurs when the system is fast growing and very vulnerable to fire, in other words, when both dynamics (with and without fire) are strongly attractive to their steady states. Assuming that all the couples 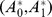 in Fig. 2a are equiprobable, in 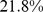 of the parametric configurations the tree cover distribution is bimodal, and in 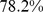 unimodal.

If the fire probability in the disturbed steady state is high 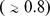, the ecosystem tends to be frequently burned, 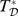 has a low risk to be left, the dynamics with fire are more frequent, and the woody cover is in average low (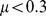). If the fire probability is low in both steady states (the bottom corner of Fig. 2b), the dynamic without fire is more frequent, 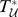 has a low risk to be left and the woody cover is in average high (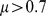). Bimodality occurs when the fire probability is very low in 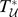 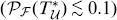 and relatively high in 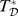, 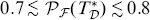, so that the dynamics with fire tend to be maintained when the ecosystem is in 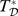, and the dynamics without fire tend to be maintained when the ecosystem is in 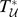. Assuming that all the couples 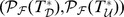 in panel b) of Fig. 2 are equiprobable, in 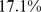 of the parametric configurations the tree cover distribution is bimodal and in 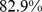 unimodal. In general, the tree cover tends to be bimodal when both steady states have a low risk to be left.

The second example shows that the operation of spatial sampling of tree cover can lead to a bimodal frequency distribution. Data are considered as collected from different sites. A bimodal frequency distribution is obtained if data are collected from sites having (Fig. 3a) all bimodal probability distributions, or alternatively, if one samples from sites (Fig. 3b) having unimodal probability distribution but with both high and low values of the tree cover, *i.e.*, sampling from parameter configurations corresponding to both the top and the bottom right corner of Fig. 2a.

The third example illustrates the presence of rapid transitions between moist savanna and tropical forest, as in [17]. The analysis of the trajectory in Fig. 4 leads to some considerations. It is possible to see how 1) for 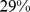 of time the ecosystem is characterized by a total tree cover 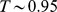, assimilable to a forest state, and for 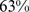 of time by 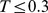, assimilable to an open savanna state. The forest and savanna states are observable for long periods, see respectively the intervals 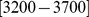 and 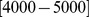 in Fig. 4a. 2) The feedbacks between fire and woody cover are evident through the clustering of fire events (see Fig. 4b). 3) Fire-tree feedbacks allow sharp (but not catastrophic) transitions forest→savanna and savanna→forest (Fig. 4a). 4) The stationary probability distribution of the total tree cover is bimodal (Fig. 4c).

Why should this alternative explanation to tree cover bimodality be plausible?

Our explanation of tree cover bimodality stems from a characteristic element of the moist savannas and tropical forest dynamics: the fire occurrence is not constant, but is a variable, dependent on the ecosystem status, with a feedback between fire and trees [9], [10]. If this phenomenological issue is quite evident, the simple models available in literature, *i.e.*, space-implicit ordinary differential equation models, see e.g. [4]–[6] ignore this element. In particular, these models are non-linear, and assume the fire frequency as a constant parameter. As a consequence, these models lead to bistability between moist savanna and forest. Here, differently, we include the variability of fire occurrence in a matrix model of tree dynamics, where the impact of fire is of on-off type and depends on the tree cover, driving the ecosystem to oscillate stochastically between two dynamics, each one characterized by one steady state. In this way, the relative strength of the two dynamics can determine whether the tree cover is unimodal or bimodal.

## Conclusions

Alternative stable states, tipping points, catastrophic transitions, and early warnings are recurrent issues in many ecological dynamics, and the tree cover variability of moist savannas and tropical forests is not an exception [1], [2]. However, evidences of catastrophic transitions and early warnings of tipping points in natural ecosystems are still elusive [13], [39]. Here we have started our analysis from the observed bimodal frequency distribution of tree cover in moist savannas and tropical forests, which is considered a proof of savanna/forest bistability [1], [2]. We have presented an alternative explanation to the bimodal frequency distribution of tree, which does not require alternative stable states and corresponding catastrophic transitions. Because in these ecosystems fire is one of the main determinants of the vegetation dynamics, dependent on the ecosystem state, and with different impact depending on the tree height, we have used a matrix model to represent the yearly dynamics of trees, considering a matrix when fire occurs, and another matrix when fire does not occur, with feedbacks between fire and trees. We have found that 1) the switching between the two tree dynamics, one with and one without fire, with fire-tree feedbacks, may bring out a bimodal stationary probability distribution of tree cover. The matrix model can be assimilated to a Markov chain allowing to determine the stationary probability distribution of tree cover, which can be interpreted as the distribution of the relative frequency of the visits in each state along a simulation. 2) The spatial sampling can facilitate the observation in frequency of tree cover bimodality. 3) The feedbacks between fire and woody cover are included in the dynamics without necessarily having alternative stable states, contrary to what reported in literature [13]. 4) Sharp transitions between savanna and forest are possible, but these are not necessary catastrophic in the system dynamics sense. This change of perspective about the tree cover bimodality could have profound implications in the management of wet savanna and tropical forest ecosystems. In addition, switching mechanisms between different dynamics could be useful to explain the existence of other emerging behaviors, like the formation of vegetation patterns, as depicted by [40] and [41], or to clarify the vegetation transitions in other ecosystems, for example understanding the findings of [42] in drylands.
